# Impact of information framing and vaccination characteristics on parental COVID-19 vaccine acceptance for children: a discrete choice experiment

**DOI:** 10.1007/s00431-022-04586-6

**Published:** 2022-09-02

**Authors:** Kailu Wang, Eliza Lai-Yi Wong, Annie Wai-Ling Cheung, Vincent Chi-Ho Chung, Charlene Hoi-Lam Wong, Dong Dong, Samuel Yeung-Shan Wong, Eng-Kiong Yeoh

**Affiliations:** 1 Centre for Health Systems and Policy Research, JC School of Public Health and Primary Care, Faculty of Medicine, The Chinese University of Hong Kong, Hong Kong, China; 2 The Jockey Club School of Public Health and Primary Care, Faculty of Medicine, Prince of Wales Hospital, The Chinese University of Hong Kong, Room 418, 4/F, Hong Kong, China

**Keywords:** Vaccine hesitancy, Preference, Conjoint analysis, Message framing, Loss frame

## Abstract

This study aimed to test the influence of vaccination characteristics and gain/loss-framing of information, on parental acceptance of the coronavirus disease 2019 (COVID-19) vaccination for their minor children. A discrete choice experiment was conducted among parents of children aged 0–17 years from September to October 2021 in Hong Kong. Respondents were randomly assigned to four groups with different framing of information and asked to choose hypothetical vaccination alternatives, described by seven attributes that were derived from prior qualitative interviews. A mixed logit model was adopted to analyze the effect of attributes and information framing on parental vaccination acceptance. The vaccine acceptance rates under different scenarios were also estimated. A total of 298 valid responses were obtained. It was found that the BioNTech brand, higher efficacy, less serious adverse events and more vaccination coverage in children significantly improved parental acceptance. Additionally, loss-framing increased parental acceptance compared with gain-framing, while the presentation of mortality information did not make a difference. Acceptance was also associated with parental uptake of the COVID-19 vaccine and the children’s age.

*Conclusion*: The findings imply that factors including gain/loss information framing, importance of vaccine characteristics, and peer influence have a significant effect on parents’ decisions to get their children vaccinated. Parents with younger children had greater vaccine hesitancy, and information framing techniques should be considered in vaccination promotion for combating such vaccine hesitancy. Future studies could be conducted to identify the moderators and mediators of information framing to facilitate its implementation.

**Table:** 

**What is Known:** *• Parental acceptance of COVID-19 vaccine was found to be associated with various socio-economic and psychosocial factors, while the evidence on impact of vaccination characteristics was limited.* *• Behavioral interventions, including information framing, have been used to promote various health behaviors.*	
**What is New:** *• Loss-framing of information on vaccine effectiveness improves vaccine acceptance, while additional information on how the vaccine reduces death does not make a difference, which can be used to inform communication with the public in vaccination promotion.* *• The social norm (i.e., the vaccine uptake amongst other people) is important for increasing the parental vaccine acceptance rate.*

**Supplementary Information:**

The online version contains supplementary material available at 10.1007/s00431-022-04586-6.

## Introduction

Among the population, the risk of Coronavirus disease 2019 (COVID-19) infection in children and adolescents under 18 years should not be overlooked. In the USA, approximately 10.9% of children under 18 years of age were found to be seropositive for SARS-CoV-2 antibody from May to September 2020 [1]. To reduce the infection rate among children and adolescents, vaccination is one of the most notable ways found to be even more effective among adolescents than adults in clinical trials and real-world data [2, 3] — this despite the fact that they received vaccination a few months later than adults [4]. Therefore, increasing the level of vaccination acceptance among children under 18 years of age is essential to control disease spread.

However, the hesitancy of adults to vaccinate their children has been reported in previous studies on various diseases [5, 6]. A number of studies have also been conducted to identify the individual-level factors associated with parental vaccine acceptance of COVID-19 and influenza, which included previous vaccinations of the adult, educational attainment, household income, children’s age, and perceived safety and efficacy of the vaccines [7, 8, 9, 10, 11, 12]. However, relatively few studies have identified vaccine characteristics that affect COVID-19 vaccine acceptance for children, rather than socioeconomic and psychosocial factors.

Apart from the impact of vaccine characteristics, behavioral interventions, including framing of vaccine-related information, have been considered for vaccination promotion [13]. Among them, the gain/loss frame is one of the common types of framing derived from prospect theory, which demonstrates that people are more sensitive to loss than gain of the same magnitude [14]. It is also one of the intervention approaches known as “nudge,” which aims to create or change the environment where people make decisions while maintaining their freedom of choice [15, 16]. In gain-/loss-framing, the message emphasizes the benefit (gain) or cost (loss) associated with a certain behavior [17]. In previous studies, gain-framing was found to be more effective in promoting behaviors, including the use of sunscreen or physical exercise, while loss-framing was more persuasive in detection behaviors, including colonoscopy [18]. Their effectiveness is also associated with the level of perceived susceptibility to a health condition and potential risk of the behaviors, where gain-framing is more effective in low-risk behavior for low-susceptibility conditions or in “promotion-focused” appeals, while loss-framing is more effective in behaviors with higher risk for high-susceptibility conditions or in “preventive-focused” appeals [17, 19, 20, 21]. In vaccinations against other diseases, a systematic review summarized that different types of nudging, including change in default option, offering incentives, and change of messengers could motivate people to get vaccinated [22]. Text reminders stated that the influenza vaccine is “waiting for you” could also improve the vaccine uptake in pharmacies [23]. Meanwhile, change of message framing showed mixed results, as some studies found loss-framing promoted human papillomavirus (HPV) or measles, mumps, and rubella vaccines [24, 25], while others found no effect of gain/loss-framing on HPV and influenza vaccinations [26, 27] or improvement of gain-framed messages in vaccination intention among future-minded women [28]. For COVID-19 vaccination, a recent study in Japan found a loss-framed message about the influence of one’s vaccination behaviors on other people, could strengthen the intention to get vaccinated [29]. No studies, however, have yet tested the impact of gain/loss-framing on COVID-19 vaccination among children.

In Hong Kong, the vaccine hesitancy rate among adults maintained at approximately 40–60% in early 2021 and dropped to 20–30% after August 2021 [30]. For parental vaccination for children aged 5–11 years, approximately 70–80% of parents reported that they were unwilling to vaccinate their children in January 2022, which is higher than many other countries and regions worldwide [31]. By the time of the study, 57% of the adults and 55% of adolescents received two doses of vaccines [32]. While the government aimed to boost the vaccination rate to 70–90%, no laws or regulations associated vaccination and social distancing or other measures for the general public or children have been implemented, while a few occupation groups such as healthcare workers are required to receive vaccination. In order to improve the vaccine uptake of children and adolescent, effective interventions should be devised to promote parental vaccine acceptance for their children.

To develop an effective COVID-19 vaccine implementation strategy among children, this study aimed to test the influence of both vaccination characteristics and gain-/loss-framing on parental acceptance of COVID-19 vaccination for their minor children. Presentation of vaccine effectiveness in terms of mortality reduction was also added, as it might increase the perceived effectiveness of the vaccine and potential loss associated with non-vaccination. It was hypothesized that (1) the characteristics of the vaccine and vaccination arrangements would affect parental acceptance; and (2) the gain-/loss-framing of information on vaccination and presentation of mortality information would have different effects on parental acceptance.

## Methods

### Study sample and data collection

A discrete choice experiment (DCE) was conducted among parents with children aged 0–17 years, along with a cross-sectional questionnaire survey during mid-September and the end of October 2021 in Hong Kong (HK). This study targeted Chinese parents with minor-aged children in HK. Parents who were living in HK and had at least one child aged 0–17 years were eligible for the study, while those who were not living in HK were excluded. The participants were invited to participate in our previous surveys [33, 34]. This panel comprises a well-stratified sample of working-age adults (18–64 years) living in HK. Invitations with a link to the web-based survey were sent through text messages to these adults to introduce the study and eligibility criteria, which included being parents of at least one minor child. The survey, including the DCE, was self-administered, with two trained staff available on the instant messaging software and phone to answer inquiries from study participants. The questionnaire was conducted in Chinese. People who agreed to participate in the survey were provided a link to the formal consent form and the survey questions. Screening questions for eligibility were asked at the beginning of the survey, and another question regarding the number of minor children was again posed prior to the questions for DCE for confirmation and validation purposes. As this study involves presentation of hypothetical information, statements highlighting the information presented in the study is hypothetical were shown before and after the study, and in all the DCE choice sets.

### DCE attributes and levels

The attributes and levels of the DCE for vaccination of the oldest minor child were designed based on a prior qualitative study [35] and a literature review (details in supplementary file). As the qualitative study aimed to find out the adults’ view for their own vaccination, we conducted an additional literature review to identify the attributes that are more relevant to children’s vaccination. Combining the results of qualitative studies and literature review, the attributes for this DCE included the vaccine brand, efficacy, probability of serious adverse events, vaccine uptake among acquaintances’ minor children, vaccination coverage among all children under 18 years, recommendation from professionals, and venue for vaccination (Table 1). The vaccine brand was included as an attribute in addition to efficacy and safety, as it is considered an important factor in the qualitative study, and can help us to determine the preference for a brand and the efficacy/safety independently from each other.

**Table 1:** The attributes and levels of the DCE and information framing

DCE attributes	Levels
Brand	Sinovac (level 1); BioNtech/Pfizer/Fosun (level 2).^a^
Efficacy	50% (level 1); 70% (level 2); 90% (level 3);
Probability of serious adverse event	1 in 10,000 (level 1); 1 in 100,000 (level 2)
Vaccine uptake among acquaintances’ minor children	None received the vaccine (level 1); Some children in relative/friends’ family received the vaccine (level 2)
Vaccination coverage among all children under 18 years	20% (level 1); 40% (level 2); 60% (level 3)
Recommendations from professionals	Recommended by the general physicians/pediatricians (level 1); Recommended by the expert panel of the government (level 2)
Venue for vaccination	Community hall (level 1); Housing estate/school (level 2); Healthcare facilities (level 3)
**Gain/loss-framing of information and presentation of mortality information**	
Gain-frame + no mortality	If a child is vaccinated, he/she has around 1/2—1/20 probability of COVID-19 infection of un-vaccinated people
Gain-frame + mortality	If a child is vaccinated, he/she has around 1/2—1/20 probability of COVID-19 infection of un-vaccinated people, and has around 1/7–1/30 risk of death of un-vaccinated people
Loss-frame + no mortality	If a child is **not** vaccinated, he/she has around 2–20 times probability of COVID-19 infection of vaccinated people
Loss-frame + mortality	If a child is **not** vaccinated, he/she has around 2–20 times probability of COVID-19 infection of vaccinated people, and has around 7–30 times risk of death of vaccinated people

^a^Refer to as “BioNtech” in the text and below tables

### Experimental design

Prior to the formal tasks, background information on the COVID-19 vaccine was provided to the respondents. In addition, each individual was randomly allocated to one of the different information groups presenting the expected outcome of the vaccine (Table 1) — stratified by age and sex — which were designed based on previous studies [18, 20]. The first information involved the gain frame versus the loss frame of the information on the vaccine outcome. The second involved showing or not showing the expected outcome of reducing mortality, in addition to infection information. The framing of mortality information followed the way of framing infection in the first block, so that each respondent would only access either the gain frame or loss frame of the information. The figures used in the information were obtained from a previous study on both selected vaccines [36, 37].

The design of the choice set was optimized based on D-efficiency with zero prior means on the main effects in Stata 15.0. A total of 32 pairwise choice sets were selected from the full factorial design of the attribute levels. To reduce the cognitive burden of the respondents, these choice sets were randomly assigned to four blocks with eight choice sets in each. An opt-out option for “do not accept vaccination in 6 months” was added to each choice set to record vaccine refusal behaviors. The attribute order and the choice task sequence in each block were randomized at the individual level, and the alternative order was randomized for different choice tasks. An example of the presentation of information and choice tasks is shown in Table 2.

**Table 2:** Example of the choice sets in the survey

**Hypothetical scenario (repeated at every choice task):** a) Both Sinovac and BioNtech have been tested in scientific research that they can be used, and approved by the government, in children and adolescent under 18 years. Its efficacy and safety in children are the same as in adults. Vaccination for children under 18 years need to be consented by their parents. The vaccination is free-of-charge. They can get the vaccination with or without prior online reservation, and with or without company of the parents. The other vaccination procedures are the same as the adults b) Please note that: If a child is **not** vaccinated, he/she has around 2–20 times probability of COVID-19 infection of vaccinated people			
	Vaccination plan 1	Vaccination plan 2	No vaccination for 6 months
Brand	BioNTech/Pfizer/Fosun	Sinovac	No vaccination for 6 months: **□**
Efficacy *	90%	70%	
Probability of serious adverse event *	1 in 10,000	1 in 100,000	
Vaccine uptake among acquaintances’ minor children	Some children in relative/friends’ family received the vaccine	None received the vaccine	
Vaccination coverage among all children under 18 years	40%	60%	
Recommendations from professionals	Recommended by the general physicians/pediatricians	Recommended by the expert panel of the government	
Venue for vaccination	Community hall	Healthcare facilities	
*The above information is hypothetical and for research purposes only. For the actual details, please refer to the information provided by the Department of Health			
Which vaccination plan would you like to choose?	Vaccination plan 1: **□**	Vaccination plan 2: **□**	

### Study questionnaire

Besides the DCE tasks, the study questions were designed based on previous research on vaccination and the health belief model [38, 39], which included: (1) perception of oneself and experience during the pandemic, perceived risk and perceived severity of COVID-19 infection, and worries over being quarantined due to being in close contact with confirmed cases, and whether they received vaccination; (2) perception of the oldest minor child, including perceived risk of COVID-19 infection, worries over side effects for the children, perceived effectiveness of the vaccination, worries over missing work/school due to the child’s infection, and a multiple choice question for the intention to vaccinate this child; and (3) socio-economic status of the respondents and the age of oldest child. These questions were located before the information blocks and DCE tasks. Cronbach’s alpha for the perception scales was 0.734, which indicated an acceptable and relatively high internal consistency of the questions [40]. Prior to the survey, six public health professionals and ten adults were invited to participate in a pilot survey to provide feedback and an assessment of the questionnaire, including the DCE, to improve content validity.

### Statistical analysis

The data analysis was divided into two steps. First, a multiple logistic model was used to examine the association between the intention to vaccinate the children and individual-level characteristics of the parents and children. The independent variables were selected according to the outcomes of previous studies on factors associated with parental opinions on children’s vaccination. The dependent variable was the dichotomous-choice question on vaccination acceptance for children (yes/no). Second, as utility (U) for accepting the vaccine for children consists of a deterministic component (V) and a stochastic component (ε) — where the deterministic component can be considered as a function of utility derived from the attribute levels of vaccination alternatives in the DCE — utility can be estimated as [41]:

$$\begin{aligned}{U}_{i}={V}_{i}+ {\upvarepsilon }_{i}&\\{V}_{i|opt-in} &={{\beta }_{0}+\beta }_{1}\cdot brand\left(BNT\right)+ {\beta }_{2}\cdot efficacy\left(70\%\right)+\\&{\beta }_{3}\cdot efficacy\left(90\%\right)+ {\beta }_{4}\cdot SAE\left(1 in \mathrm{100,000}\right)+\\&{\beta }_{5}\cdot acquaintance\left(yes\right)+ {\beta }_{6}\cdot coverage\left(40\%\right)+\\&{\beta }_{7}\cdot coverage\left(60\%\right)+{\beta }_{8}\cdot recommend\left(government\right)+\\&{\beta }_{9}\cdot venue\left(housing/school\right)+{\beta }_{10}\cdot venue\left(healthcare\right)\\{V}_{i|opt-out }=0&\end{aligned}$$

As the utility cannot be measured directly, the dichotomous response to each DCE alternative (chosen/not chosen) was used as the dependent variable in the model. Stata 15.0 was utilized in data analysis, and a mixed logit model (MIXL) was adopted to examine the influence of DCE attribute levels on vaccination intention for children, considering the unobserved preference heterogeneity across individuals (Stata command: mixlogit). The coefficients (β) represent utility weightings of the attribute levels of a vaccine. Opt-in for vaccination was considered as an alternative-specific constant (ASC, β_0_) in the model, considering its interactions with the two randomized information blocks (i.e., gain/loss information framing, and mortality information) for examining the effect of information presentation on vaccine acceptance. The model specification, including interaction terms, was taken as reference from an introduction of mixed logit model [42]. The individual-level factors that were found to be significantly associated with vaccine acceptance for children in the first step were also added to MIXL as interactions with opt-in to determine differences in the acceptance of DCE questions among these subgroups. The acceptance rate of vaccines with different characteristics and under different scenarios, were further estimated using the regression outcome as exp(V)/(1 + exp(V)), where the V is the sum of regression coefficients β of related independent variables [43].

## Results

### Respondent characteristics

A total of 1615 people aged 18–64 years who previously agreed to be followed-up on COVID-19–related survey were approached in the study, and 1299 agreed to participate. The response rate was 80.4%. Among them, 298 (22.9%) reported having at least one minor child, which is a similar proportion to the statistics provided by the census that 23.1% of households in HK have one or more minor children [44]. Within this group, 3.0% were aged below 24 years, 60.4% between 25 and 44 years, and 36.6% between 45 and 64 years, which is also similar to the age distribution of household heads with one or more children (1.1%, 58.5%, and 40.4% for these three age groups, respectively) [44]. In the sample size estimation, with reference to approximately 70% parental acceptance rate found in a Chinese population in the city next to HK [9], the minimum sample size for DCE with eight choice sets, 4% accuracy level for each respondent, and 5% significant level should be 129, using the estimation formula provided by a previous DCE study [45]. Therefore, 298 participants were sufficient for the analysis. The sample characteristics are listed in Table 3. No significant differences in respondent demographics and psychosocial characteristics were found across the different information groups (Supplementary Table S2). A description of response behaviors can be found in the supplementary file.

**Table 3:** Respondent’s characteristics according to parental vaccination intention

	Total		Parental vaccination intention^1^				*P* values
			Not intended		Intended		
	**N**	%	**N**	%	**N**	%	
**Age of parents**							
18–34 yrs	82	27.5	12	40.0	70	26.1	0.163
35–49 yrs	170	57.1	16	53.3	154	57.5	
50 + yrs	46	15.4	2	6.7	44	16.4	
**Sex**							
Male	114	38.3	7	23.3	107	39.9	0.076
Female	184	61.7	23	76.7	161	60.1	
**Education**							
Below bachelor degree	161	54.0	14	46.7	147	54.9	0.394
Bachelor degree or above	137	46.0	16	53.3	121	45.2	
**Household income**							
Below HK$30,000	126	42.3	8	26.7	118	44.0	0.068
HK$30,000 +	172	57.7	22	73.3	150	56.0	
**Chronic conditions of parents**							
No	245	82.2	24	80.0	221	82.5	0.738
Yes	53	17.8	6	20.0	47	17.5	
**Parental uptake of COVID-19 vaccine**							
No	58	19.8	20	69.0	38	14.4	< 0.001*
Yes	235	80.2	9	31.0	226	85.6	
(missing)	5		1		4		
**Perceived “likely/very likely” to be infected**							
No	188	63.1	21	70.0	167	62.3	0.408
Yes	110	36.9	9	30.0	101	37.7	
**Perceived “slightly severe/very severe” if get infected COVID-19**							
No	143	48.0	19	63.3	124	46.3	0.076
Yes	155	52.0	11	36.7	144	53.7	
**“Slightly/very” worry about being quarantined**							
No	90	30.2	14	46.7	76	28.4	0.038*
Yes	208	69.8	16	53.3	192	71.6	
**Age of children**							
0–4 yrs	78	26.2	13	43.3	65	24.3	0.024*
5–11 yrs	98	32.9	11	36.7	87	32.5	
12–17 yrs	122	40.9	6	20.0	116	43.3	
**Perceived the children “likely/very likely” to be infected**							
No	80	27.3	14	48.3	66	25.0	0.008*
Yes	213	72.7	15	51.7	198	75.0	
(missing)	5		1		4		
**Perceived effectiveness of the vaccine for children**							
Low	128	43.0	21	70.0	107	39.9	0.007*
Medium	119	39.9	6	20.0	113	42.2	
High	51	17.1	3	10.0	48	17.9	
**“Slightly/very” worry about vaccine side effect for children**							
No	77	26.3	3	10.3	74	28.0	0.040*
Yes	216	73.7	26	89.7	190	72.0	
(missing)	5		1		4		
**“Slightly/very” worry about missing school or work due to children’s infection**							
No	132	45.1	17	58.6	115	43.6	0.122
Yes	161	55.0	12	41.4	149	56.4	
(missing)	5		1		4		
**Total**	298	100.0	30	100.0	268	100.0	

*P < 0.05; 1. the parental vaccination intention shown in the table were elicited using a multiple choice question

### Influence of DCE attributes and information framing on vaccination likelihood

Table 4 shows the MIXL results on the influence of the DCE attributes and information framing. For the attribute levels, the respondents preferred BioNTech (coefficient [coeff]: 0.65, 95%CI: 0.42–0.89), higher vaccine efficacy (70% efficacy: coeff: 0.33, 95%CI: 0.09–0.56; 90% efficacy: coeff: 0.70, 95%CI: 0.43–0.97), lower serious adverse event rate (coeff: 0.37, 95%CI: 0.16–0.57), and higher overall coverage among minor children in HK (40% coverage: coeff: 0.33, 95%CI: 0.11–0.55; 60% coverage: coeff: 0.39, 95%CI: 0.13–0.64). For information framing, a loss frame of information on the effectiveness of the vaccine can improve the likelihood of accepting vaccination compared with the gain frame (coeff: 1.04, 95%CI: 2.02–0.07); while presentation of the effect of vaccine on reducing mortality did not significantly change the vaccine acceptance for children (coeff: 0.11, 95%CI: −0.80–1.03). Supplementary analysis found that people in the loss frame group rated vaccine safety less important than the gain frame in DCE (loss frame: 0.23, 95%CI: −0.06–0.52; gain frame: 0.58, 95%CI: 0.29–0.86) (Supplementary Table S3).

**Table 4:** Effects of vaccination characteristics and information framing on parental vaccine acceptance for children’s COVID-19 vaccination in DCE

		Coefficient	95%CI^1^
*Mean*			
**Brand (Sinovac as reference)**			
BioNtech (β_1_)		0.65**	(0.42, 0.89)
**Efficacy (50% as reference)**			
70% (β_2_)		0.33*	(0.09, 0.56)
90% (β_3_)		0.70**	(0.43, 0.97)
**Serious adverse event (1/10,000 as reference)**			
1/100,000 ppl (β_4_)		0.37**	(0.16, 0.57)
**Vaccine uptake among acquaintances’ minor children (None as reference)**			
Some children received the vaccine (β_5_)		0.08	(−0.10, 0.26)
**Vaccination coverage among all children under 18 years (20% as reference)**			
40% (β_6_)		0.33*	(0.11, 0.55)
60% (β_7_)		0.39*	(0.13, 0.64)
**Recommendations from professionals (government experts as reference)**			
Physician/pediatricians (β_8_)		−0.01	(−0.19, 0.18)
**Venue for vaccination (community hall as reference)**			
Housing estate/school (β_9_)		0.00	(−0.24, 0.25)
Healthcare facilities (β_10_)		0.21	(−0.03, 0.45)
**Opt-in (ASC**^**1**^**,** β_0_**)**		−4.70**	(−6.26, -3.13)
**Loss frame x opt-in (**β_lossframe_**)**		1.04*	(0.07, 2.02)
**Mortality x opt-in** (β_mortality_)		0.11	(−0.80, 1.03)
**5–11 years x opt-in** (β_5-11 yr_)		0.66	(−0.25, 1.57)
**12–17 years x opt-in** (β_12-17 yr_)		1.79**	(0.84, 2.75)
**Parent uptake of vaccine x opt-in** (β_parent_)		1.71**	(0.85, 2.58)
*Standard deviation*			
**Brand (Sinovac as reference)**			
BioNtech		1.80**	(1.54, 2.05)
**Efficacy (50% as reference)**			
70%		0.89**	(1.19, 0.58)
90%		1.19**	(0.89, 1.50)
**Serious adverse event (1/10,000 as reference)**			
1/100,000 ppl		1.24**	(1.03, 1.45)
**Vaccine uptake among acquaintances’ minor children (None as reference)**			
Some children received the vaccine		0.37*	(0.65, 0.10)
**Vaccination coverage among all children under 18 years (20% as reference)**			
40%		0.40*	(0.17, 0.62)
60		1.07**	(0.78, 1.35)
**Recommendations from professionals (government experts as reference)**			
Physician/pediatricians		0.45**	(0.23, 0.67)
**Venue for vaccination (community hall as reference)**			
Housing estate/school		0.34	(−0.02, 0.71)
Healthcare facilities		0.14	(−0.40, 0.67)

*P < 0.05; **P < 0.001; 1. *CI*, confidence interval; *ASC*, alternative specific constant

The parental vaccine acceptance rate for minor children was predicted using estimates from the MIXL model (Supplementary file, Figs. S1, S2, S3 and S4). The highest acceptance rate (91.4%) was found for the BioNtech vaccine (approximately 90% efficacy) [37] for children aged 12–17 years, and with parents who received the vaccine and received information on the effectiveness of the vaccine in both disease incidence and mortality in loss-framing messages, when other attributes were considered at their optimal levels. This probability is estimated as exp(2.36)/(1 + exp(2.36)), where 2.36 = β_lossframe_ + β_mortality_ + β_12-17 yr_ + β_parent_ + β_0_ + β_1_ + β_3_ + β_4_ + β_5_ + β_7_ + β_8_ + β_10_, as shown in Table 4. The acceptance rate for Sinovac (approximately 70% efficacy) [36] was approximately 10–20 percentage points (PP) lower than that for BioNtech. The loss frame for the information increased approximately 10 PP regarding vaccine acceptance, while the presence of mortality information made little difference. The acceptance rate for 5–11-year-old children with parents who received the vaccine was approximately 20 PP lower than 12–17 years old, followed by children aged 12–17 years old (30–40 PP lower) and 5–11-year-old children (50–60 PP lower) with parents who did not receive the vaccine.

## Discussion

This study addressed the research gap on the influence of information presentation or framing on parental acceptance of COVID-19 vaccine for children and adolescents, by showing that the loss framing of vaccine efficacy information improves parental acceptance of the vaccine, which has not previously been tested for COVID-19 vaccination among children as far as we know. It also examined the influence of vaccination characteristics on parental acceptance, and found that the vaccine brand, efficacy, safety, and vaccination coverage affect parental vaccine acceptance for children, and associations between vaccine acceptance and uptake of vaccine by the parents as well as the age of the children.

From DCE findings, nearly one-fifth of respondents constantly refused vaccination alternatives, which is slightly lower than the rate of vaccination refusal found for themselves (23.6%) uncovered at the beginning of the vaccination program [38]. Among the attributes, the influence of vaccine efficacy, brand, and safety was similar to the outcomes found for COVID-19 vaccine acceptance [38, 43] among adults, and parental preference for any vaccine for children [46, 47]. The vaccine brands also had a large effect independent of their efficacy and safety indicators, which may reflect its reputation, place of origin, type of technologies, and reported incidents associated with the brands. In addition, the overall coverage of vaccination among the minor children population can also affect acceptance. Comparable finding from a previous study on parental preference showed that population coverage had a greater effect than coverage among acquaintances [47]. These findings suggest the importance of peer influence or social norms in promoting vaccine acceptance; whereas the free-rider effect, which may result in a higher vaccine hesitancy rate when the vaccination coverage is higher, is unlikely to be found.

However, these factors are not easily modified. Framing information can be considered a way to communicate with the public or specific target groups in vaccination promotion. The study results suggested that more parents would accept vaccines for their children when receiving loss-framing messages of vaccine effectiveness than receiving a gain-framing message. A possible reason for this may be that when people are facing potential gains of vaccination (gain-framing), they do not feel that the gains are large enough to exceed the potential loss if their children undergo the risk or side effects of vaccination; therefore, some of them would rather choose no vaccination without any gain or loss. On the contrary, when facing potential loss in non-vaccination (loss-framing), this loss was perceived to be higher than the potential loss (risk or side effects) of vaccination as people tend to be loss-aversive, and therefore they would rather have their children vaccinated [14, 18]. Supplementary analysis also revealed that people receiving loss-framing information considered vaccine safety risk to be less important in decision-making. These findings are consistent with a study in mainland China that loss-framing information had a higher impact on vaccination intentions than gain-framing information for adults [20]. However, the effect of gain/loss framing was rarely found in influenza and hepatitis vaccinations [27, 48, 49]. This may be because COVID-19 vaccination for children was initiated in less than half a year; therefore, parents are likely to have uncertainty regarding its safety and hence have a higher perceived risk of “losses” for children’s vaccination than other vaccines [10].

When adopting information framing in vaccination promotion, there are a few factors that need to be considered for implementation. Psychological uncertainty, which refers to the level of people’s insecurity around one’s knowledge, may influence the effects of gain/loss framing. The loss frame of information was found to be more persuasive among people with a higher level of uncertainty than low uncertainty [49], as it may lead to perceived threats to freedom and psychological reactance for people with low uncertainty, as they might be aware of the manipulation and persuasive attempts. Moreover, the loss frame was found to be more likely to trigger fear and powerlessness among the information recipients [50], which on the one hand led to compliance and acceptance of vaccination and strict policies in containing COVID-19, and on the other hand, may lead to mental health issues such as anxiety. Therefore, more individualized framing interventions should be considered, rather than massive campaigns at the population level.

Some limitations in this study, however, should be noted. First, the DCE did not incorporate graphics to illustrate the attribute levels, as the format of the tasks did not present well in mobile devices, which most respondents used to answer the questionnaire. Second, information framing may seem to be a duplicate of the attribute of efficacy; however, the attribute of efficacy was used as a standard presentation where only the percentages were shown, while the background information blocks adopted text to frame the meaning of the numbers. The range of effectiveness used in information framing (e.g., 1/2–1/20 or 2–20 times) also covers the range of all attribute levels. Third, caution should be taken in the design and implementation of study that involves hypothetical messages and misinformation for respondents (e.g., unreal combination of vaccine brand and efficacy), which may expose them to incorrect information that may affect their future behaviors. Although this study provided a statement in each of the choice sets to highlight the hypothetical nature of the information shown in the choice tasks (Table 2) and reduce risk of misunderstanding, use of misinformation in future studies should be minimized for topics closely related to human health. Even if the misinformation components cannot be avoided or substituted by another method, it should be exercised prudently, where there should be emphasis on the nature of hypothetical information in both verbal and written statements in the study, a screening procedure for participants with ability to distinguish the accuracy of information, and a timely and comprehensive debriefing session and follow-up communications in place to help respondents receive correct information in the study, among other measures to reduce the potential adverse consequences.

## Conclusions

This study revealed that vaccine characteristics and coverage in children affected parental acceptance of the vaccine, and that the gain/loss-framing effect of information could also make a difference. The findings indicated the importance of parental vaccine acceptance and children’s age in the decisions that parents take on whether to vaccinate their children. It also implied that peer influence or social norm influence parental decisions on whether to vaccinate their children, while freeriding was not found. Meanwhile, authorities and healthcare organizations should consider adopting information framing techniques to promote vaccination. Moreover, there is a need for further studies focusing on the potential moderators and mediators of information framing, to increase vaccine acceptance.

## Supplementary Information

Below is the link to the electronic supplementary material.

Supplementary file1 (DOCX 78 KB)

## Data Availability

Data used in this study will be available upon reasonable request.

## Code availability

The code will be available upon reasonable request following publication.

## Abbreviations

AOR: Adjusted odds ratio
ASC: Alternative-specific constant
CI: Confidence interval
Coeff: Coefficient
COVID-19: Coronavirus disease 2019
DCE: Discrete choice experiment
HK: Hong Kong
HPV: Human papillomavirus
MIXL: Mixed logit model
PP: Percentage point
