# Supplementary material for: Impact of information framing and vaccination characteristics on parental COVID-19 vaccine acceptance for children: a discrete choice experiment
Source: Eur J Pediatr. 2022 Sep 2;181(11):3839–49. doi: 10.1007/s00431-022-04586-6 (PMC9439717; doi:10.1007/s00431-022-04586-6)
Supplement: Supplementary file 1 — Supplementary file1 (DOCX 78 KB) [file 431_2022_4586_MOESM1_ESM.docx]

**Supplementary materials**

1. **Qualitative interview, literature review and the attributes**

The descriptive framework was determined based on the qualitative interview results, literature review, the baseline survey conducted in Hong Kong in February 2021 (K. Wang et al., 2021), and the updated information provided by the government on its official website of COVID-19 vaccination programme (HKSAR, 2021).

1.1. Qualitative interview findings

A semi-structured individual interview was conducted among 45 adults with different age, educational level and chronic conditions for exploring the facilitators and barriers for them to be vaccinated or not vaccinated (Wong et al., 2022). It can be found from the qualitative interview results that the origins or brands of the vaccine, perceived benefits and importance of receiving the vaccination, concerns of side effects, expected resumption to normal life, suggestions from healthcare professionals and acquaintances, and travel distance to vaccination locations were more frequently reported by the participants in deciding whether or not to be vaccinated.

1.2. Literature review findings

A literature review of parental preference for children’s vaccination against COVID-19 was performed in addition to a qualitative study to identify vaccination-related characteristics that are more relevant to vaccination among children aged under 18 years. Based on these research and systematic reviews (A et al., 2021; Bishai, Brice, Girod, Saleh, & Ehreth, 2007; Gong et al., 2020; Hoogink et al., 2020; Michaels-Igbokwe, MacDonald, & Currie, 2017; Sun et al., 2020; Veldwijk, Lambooij, Bruijning-Verhagen, Smit, & de Wit, 2014; Verelst, Willem, Kessels, & Beutels, 2018; B. Wang et al., 2017), vaccination-related characteristics, including the level of protection/efficacy/effectiveness, price, risk/burden of the disease, safety/side effect, place of origin, accessibility/location or provider of vaccination, coverage of vaccination among the entire population, and coverage/uptake among acquaintances are commonly used attributes in conjoint studies for children and adolescents. Therefore, the effectiveness and risks of adverse event of vaccination, and the convenience of vaccination along with other factors identified in the qualitative interviews and literature were considered as attributes to construct the choice tasks. Vaccine price was excluded because the provision of vaccination was free of charge in HK. The risk of the disease was also excluded because this attribute was only used in vaccine DCE studies without a specific target disease, which is not suitable for this study for COVID-19 as its risk to children has been determined.

1.3. Background information and levels of the attributes

At the time of the DCE study was initiated (September 2021), a bit more than half of the adults (57%) were fully vaccinated. There were no difficulties in scheduling vaccination, due to sufficient supply and the launch of multiple vaccination centres all over the city. BioNtech vaccine has been approved for vaccination for adolescents aged 12-17 years before the survey starts, and pending for local approval for use among 5-11 year-old children. Sinovac vaccine has been approved for use in children aged 3-17 years in mainland China, and pending for approval in Hong Kong. The HKSAR government has announced the vaccination is free of charge for both adults and adolescents, while the incentives in monetary or in other forms were provided to the public who are vaccinated by the end of September by multiple local private organizations. In September 2021, two brands of vaccines were deployed in Hong Kong, and it was unlikely to introduce new vaccines within the year of 2021. All these information contributed to the hypothetical scenarios where the participants made the choices in the DCE study.

The levels of the attributes were formed based on the previous research articles, qualitative interview findings, and practice of vaccination programme in Hong Kong, and described in text in the questionnaire. The efficacy and the probability of serious adverse event of the two vaccines has been reported in the articles of phase 2/3/4 clinical trials, which were used to determine the levels of attributes (efficacy and serious adverse event) (Frenck et al., 2021; Jara et al., 2021; Polack et al., 2020; Zhang et al., 2021). The vaccine acceptance can be influenced by both the vaccine coverage in population and vaccine uptake of acquaintances including family members and friends, according to the literature review and qualitative interviews. The impact of uptake of acquaintance’s children was examined in comparison with “no vaccination of children in these family”. For population coverage, the highest level of population coverage (60%) was determined based on vaccination coverage among entire population (around 57%) in September. The other two levels of this attribute adopted a 20% interval. The recommendations for vaccination are usually made by the government expert panels and by the general physicians who have more frequent contact with the publics. The venue for vaccination has been announced to be the community halls, public hospitals/clinics and private doctors. The “residence estate/school” has been added as another attribute level as we would like to test the preference for a more convenient venue.

1. **Table S1. Respondents characteristics by different information groups**

|  | Gain + no mortality | | Gain + mortality | | Loss + no mortality | | Loss + mortality | | Total | | P value |
| --- | --- | --- | --- | --- | --- | --- | --- | --- | --- | --- | --- |
|  | N | % | N | % | N | % | N | % | N | % |  |
| **Age of parents** | |  |  |  |  |  |  |  |  |  |  |
| 18-34 yrs | 23 | 31.5 | 24 | 31.6 | 18 | 24.7 | 17 | 22.4 | 82 | 27.5 | 0.682 |
| 35-49 yrs | 42 | 57.5 | 41 | 54.0 | 43 | 58.9 | 44 | 57.9 | 170 | 57.1 |  |
| 50+ yrs | 8 | 11.0 | 11 | 14.5 | 12 | 16.4 | 15 | 19.7 | 46 | 15.4 |  |
| **Sex** |  |  |  |  |  |  |  |  |  |  |  |
| Male | 30 | 41.1 | 28 | 36.8 | 30 | 41.1 | 26 | 34.2 | 114 | 38.3 | 0.780 |
| Female | 43 | 58.9 | 48 | 63.2 | 43 | 58.9 | 50 | 65.8 | 184 | 61.7 |  |
| **Chronic conditions of parents** | | |  |  |  |  |  |  |  |  |  |
| No | 60 | 82.2 | 66 | 86.8 | 57 | 78.1 | 62 | 81.6 | 245 | 82.2 | 0.575 |
| Yes | 13 | 17.8 | 10 | 13.2 | 16 | 21.9 | 14 | 18.4 | 53 | 17.8 |  |
| **Education** | |  |  |  |  |  |  |  |  |  |  |
| Below bachelor degree | 43 | 58.9 | 36 | 47.4 | 45 | 61.6 | 37 | 48.7 | 161 | 54.0 | 0.201 |
| Bachelor degree or above | 30 | 41.1 | 40 | 52.6 | 28 | 38.4 | 39 | 51.3 | 137 | 46.0 |  |
| **Household income** | |  |  |  |  |  |  |  |  |  |  |
| Below HK$30,000 | 31 | 42.5 | 27 | 35.5 | 37 | 50.7 | 31 | 40.8 | 126 | 42.3 | 0.308 |
| HK$30,000+ | 42 | 57.5 | 49 | 64.5 | 36 | 49.3 | 45 | 59.2 | 172 | 57.7 |  |
| **Parental uptake of COVID-19 vaccine** | | | |  |  |  |  |  |  |  |  |
| No | 18 | 24.7 | 15 | 20.3 | 14 | 19.7 | 11 | 14.7 | 58 | 19.8 | 0.505 |
| Yes | 55 | 75.3 | 59 | 79.7 | 57 | 80.3 | 64 | 85.3 | 235 | 80.2 |  |
| **Age of children** | |  |  |  |  |  |  |  |  |  |  |
| 0-11 yrs | 50 | 68.5 | 45 | 59.2 | 42 | 57.5 | 39 | 51.3 | 176 | 59.1 | 0.200 |
| 12-17 yrs | 23 | 31.5 | 31 | 40.8 | 31 | 42.5 | 37 | 48.7 | 122 | 40.9 |  |
| **Perceived the children "likely/very likely" to be infected** | | | | | |  |  |  |  |  |  |
| No | 17 | 23.3 | 17 | 23.0 | 25 | 35.2 | 21 | 28.0 | 80 | 27.3 | 0.315 |
| Yes | 56 | 76.7 | 57 | 77.0 | 46 | 64.8 | 54 | 72.0 | 213 | 72.7 |  |
| **Total** | 73 | 100.0 | 76 | 100.0 | 73 | 100.0 | 76 | 100.0 | 298 | 100.0 |  |

1. **Table S2. Supplementary analysis on loss-framing’s moderator effect on relationship between vaccine safety and vaccine acceptance**

|  |  | Coefficient | 95%CI^1^ |
| --- | --- | --- | --- |
| *Mean* |  |  |  |
| **Brand (Sinovac as reference)** | |  |  |
| BioNtech |  | 0.50** | (0.27, 0.72) |
| **Efficacy (50% as reference)** | |  |  |
| 70% |  | 0.31* | (0.09, 0.54) |
| 90% |  | 0.69** | (0.43, 0.94) |
| **Serious adverse event (1/10,000 as reference)** | | | |
| 1/100,000 ppl |  | 0.58** | (0.29, 0.86) |
| **Vaccine uptake among acquaintances’ minor children (None as reference)** | | | |
| Some children received the vaccine | | -0.05 | (-0.24, 0.14) |
| **Vaccination coverage among all children under 18 years (20% as reference)** | | | |
| 40% |  | 0.18 | (-0.05, 0.41) |
| 60% |  | 0.28* | (0.02, 0.53) |
| **Recommendations from professionals (government experts as reference)** | | | |
| Physician/pediatricians | | 0.04 | (-0.14, 0.21) |
| **Venue for vaccination (community hall as reference)** | | | |
| Housing estate/school | | -0.03 | (-0.27, 0.22) |
| Healthcare facilities | | 0.16 | (-0.09, 0.41) |
| **Opt-in (ASC^1^)** |  | -2.53** | (-3.77, -1.28) |
| **Loss frame x serious adverse event** | | -0.34^2^ | (-0.74, 0.05) |
| **Loss frame x opt-in** |  | 1.51* | (0.41, 2.62) |
| *Standard deviation* |  |  |  |
| **Brand (Sinovac as reference)** | |  |  |
| BioNtech |  | 1.84** | (1.58, 2.10) |
| **Efficacy (50% as reference)** | |  |  |
| 70% |  | 0.88** | (0.65, 1.12) |
| 90% |  | 1.00** | (0.74, 1.25) |
| **Serious adverse event (1/10,000 as reference)** | | | |
| 1/100,000 ppl |  | 1.14** | (0.94, 1.35) |
| **Vaccine uptake among acquaintances’ minor children (None as reference)** | | | |
| Some children received the vaccine | | 0.83** | (0.58, 1.08) |
| **Vaccination coverage among all children under 18 years (20% as reference)** | | | |
| 40% |  | 0.86** | (0.58, 1.13) |
| 60% |  | 0.85** | (0.57, 1.13) |
| **Recommendations from professionals (government experts as reference)** | | | |
| Physician/pediatricians | | 0.28* | (0.48, 0.09) |
| **Venue for vaccination (community hall as reference)** | | | |
| Housing estate/school | | 0.29* | (0.02, 0.57) |
| Healthcare facilities | | 0.73** | (1.02, 0.43) |

Note: *P<0.05, **P<0.001; 1. CI: confidence interval, ASC: alternative specific constant. 2. For influence of serious adverse event under a loss-framing information, the coefficient should be 0.576 + (-0.344) = 0.232; the 95% confidence interval is (-0.06, 0.52), which is estimated using Delta method.

1. **Supplementary figures on estimated parental vaccine acceptance rate according to previous uptake of parents, children’s age, type of vaccine and information framing**

Figure S1. Vaccine acceptance for 12-17 years child with parents received the vaccine by information framing and vaccine type

Figure S2. Vaccine acceptance for 5-11 years child with parents received the vaccine by information framing and vaccine type

Figure S3. Vaccine acceptance for 12-17 years child with parents not received the vaccine by information framing and vaccine type

Figure S4. Vaccine acceptance for 5-11 years child with parents not received the vaccine by information framing and vaccine type

Table S3. Individual-level factors associated with parental vaccine acceptance for children

|  |  | Parental vaccine acceptance for children^1^ | |
| --- | --- | --- | --- |
|  |  | AOR^2^ | 95%CI^2^ |
| **Age of parents (18-34 yrs as reference)** | |  |  |
| 35-49 yrs |  | 0.64 | (0.18, 2.29) |
| 50+ yrs |  | 0.41 | (0.03, 5.05) |
| **Sex (Male as reference)** |  |  |  |
| Female |  | 0.31 | (0.10, 1.02) |
| **Chronic conditions of parents (No as reference)** | | |  |
| Yes |  | 0.84 | (0.19, 3.68) |
| **Education (Below bachelor degree as reference)** | | |  |
| Bachelor degree or above |  | 1.97 | (0.57, 6.85) |
| **Household income (Below HK$30,000 as reference)** | | |  |
| HK$30,000+ |  | 0.51 | (0.13, 2.01) |
| **Parental uptake of COVID-19 vaccine (No as reference)** | | |  |
| Yes |  | 19.84** | (6.34, 62.12) |
| **Perceived "likely/very likely" to be infected (No as reference)** | | | |
| Yes |  | 1.57 | (0.47, 5.23) |
| **Perceived "slightly severe/very severe" if get infected COVID-19 (No as reference)** | | | |
| Yes |  | 2.04 | (0.63, 6.63) |
| **"Slightly/very" worry about being quarantine (No as reference)** | | | |
| Yes |  | 2.41 | (0.72, 8.14) |
| **Age of children (0-4 years as reference)** | |  |  |
| 5-11 yrs |  | 2.85 | (0.74, 11.00) |
| 12-17 yrs |  | 7.81* | (1.28, 47.74) |
| **Perceived the children "likely/very likely" to be infected (No as reference)** | | | |
| Yes |  | 1.62 | (0.45, 5.87) |
| **Perceived effectiveness of the vaccine for children (Low as reference)** | | | |
| Medium |  | 2.33 | (0.70, 7.83) |
| High |  | 1.01 | (0.21, 4.75) |
| **"Slightly/very" worry about vaccine side effect for children (No as reference)** | | | |
| Yes |  | 0.12 | (0.01, 1.33) |
| **"Slightly/very" worry about missing school or work due to children's infection (No as reference)** | | | |
| Yes |  | 1.74 | (0.52, 5.80) |

Note: *P<0.05, **P<0.001; 1. the parental vaccination intention shown in the table were elicited using a multiple-choice question. 2. AOR: adjusted odds ratio, CI: confidence interval.

**Reference**

A, K., Lu, X., Wang, J., Hu, L., Li, B., & Lu, Y. (2021). Association between Adult Vaccine Hesitancy and Parental Acceptance of Childhood COVID-19 Vaccines: A Web-Based Survey in a Northwestern Region in China. *Vaccines (Basel), 9*(10). doi:10.3390/vaccines9101088

Bishai, D., Brice, R., Girod, I., Saleh, A., & Ehreth, J. (2007). Conjoint analysis of French and German parents’ willingness to pay for meningococcal vaccine. *Pharmacoeconomics, 25*(2), 143-154.

Frenck, R. W., Jr., Klein, N. P., Kitchin, N., Gurtman, A., Absalon, J., Lockhart, S., . . . Group, C. C. T. (2021). Safety, Immunogenicity, and Efficacy of the BNT162b2 Covid-19 Vaccine in Adolescents. *N Engl J Med, 385*(3), 239-250. doi:10.1056/NEJMoa2107456

Gong, T., Chen, G., Liu, P., Lai, X., Rong, H., Ma, X., . . . Li, S. (2020). Parental Vaccine Preferences for Their Children in China: A Discrete Choice Experiment. *Vaccines (Basel), 8*(4). doi:10.3390/vaccines8040687

HKSAR, G. (2021). COVID-19 vaccination programme. Retrieved from <https://www.covidvaccine.gov.hk/en/>

Hoogink, J., Verelst, F., Kessels, R., van Hoek, A. J., Timen, A., Willem, L., . . . de Wit, G. A. (2020). Preferential differences in vaccination decision-making for oneself or one's child in The Netherlands: a discrete choice experiment. *BMC Public Health, 20*(1), 828. doi:10.1186/s12889-020-08844-w

Jara, A., Undurraga, E. A., González, C., Paredes, F., Fontecilla, T., Jara, G., . . . Leon, F. (2021). Effectiveness of an inactivated SARS-CoV-2 vaccine in Chile. *New England Journal of Medicine, 385*(10), 875-884.

Michaels-Igbokwe, C., MacDonald, S., & Currie, G. R. (2017). Individual Preferences for Child and Adolescent Vaccine Attributes: A Systematic Review of the Stated Preference Literature. *Patient, 10*(6), 687-700. doi:10.1007/s40271-017-0244-x

Polack, F. P., Thomas, S. J., Kitchin, N., Absalon, J., Gurtman, A., Lockhart, S., . . . Group, C. C. T. (2020). Safety and Efficacy of the BNT162b2 mRNA Covid-19 Vaccine. *N Engl J Med, 383*(27), 2603-2615. doi:10.1056/NEJMoa2034577

Sun, X., Wagner, A. L., Ji, J., Huang, Z., Zikmund-Fisher, B. J., Boulton, M. L., . . . Prosser, L. A. (2020). A conjoint analysis of stated vaccine preferences in Shanghai, China. *Vaccine, 38*(6), 1520-1525. doi:10.1016/j.vaccine.2019.11.062

Veldwijk, J., Lambooij, M. S., Bruijning-Verhagen, P. C., Smit, H. A., & de Wit, G. A. (2014). Parental preferences for rotavirus vaccination in young children: a discrete choice experiment. *Vaccine, 32*(47), 6277-6283. doi:10.1016/j.vaccine.2014.09.004

Verelst, F., Willem, L., Kessels, R., & Beutels, P. (2018). Individual decisions to vaccinate one's child or oneself: A discrete choice experiment rejecting free-riding motives. *Soc Sci Med, 207*, 106-116. doi:10.1016/j.socscimed.2018.04.038

Wang, B., Chen, G., Ratcliffe, J., Afzali, H. H. A., Giles, L., & Marshall, H. (2017). Adolescent values for immunisation programs in Australia: A discrete choice experiment. *PLoS One, 12*(7), e0181073. doi:10.1371/journal.pone.0181073

Wang, K., Wong, E. L.-y., Cheung, A. W.-L., Yau, P. S.-Y., Chung, V. C., Wong, C. H.-L., . . . Yeoh, E. (2021). Influence of vaccination characteristics on COVID-19 vaccine acceptance among working-age people in Hong Kong, China: a discrete choice experiment. *Front Public Health*. doi:10.3389/fpubh.2021.793533

Wong, C. H., Zhong, C. C., Chung, V. C., Nilsen, P., Wong, E. L., & Yeoh, E.-k. (2022). Barriers and Facilitators to Receiving the COVID-19 Vaccination and Development of Theoretically-Informed Implementation Strategies for the Public: Qualitative Study in Hong Kong. *Vaccines, 10*(5), 764.

Zhang, Y., Zeng, G., Pan, H., Li, C., Hu, Y., Chu, K., . . . Zhu, F. (2021). Safety, tolerability, and immunogenicity of an inactivated SARS-CoV-2 vaccine in healthy adults aged 18–59 years: a randomised, double-blind, placebo-controlled, phase 1/2 clinical trial. *The Lancet Infectious Diseases, 21*(2), 181-192. doi:10.1016/s1473-3099(20)30843-4
